# Using of Multi-Source and Multi-Temporal Remote Sensing Data Improves Crop-Type Mapping in the Subtropical Agriculture Region

**DOI:** 10.3390/s19102401

**Published:** 2019-05-26

**Authors:** Chuanliang Sun, Yan Bian, Tao Zhou, Jianjun Pan

**Affiliations:** 1 College of Resources and Environmental Sciences, Nanjing Agricultural University, Nanjing 210095, China; chuanliang_sun87@163.com; 2 College of Agricultural and Economic Management, Nanjing Agricultural University, Nanjing 210095, China; bianyanlinda@163.com; 3 Department of Geography, Humboldt-Universität zu Berlin, Unter den Linden 6, 10099 Berlin, Germany; tao.zhou@ufz.de; 4 Department of Computational Landscape Ecology, Helmholtz Centre for Environmental Research—UFZ, Permoserstraße 15, 04318 Leipzig, Germany

**Keywords:** remote sensing, Sentinel-1, Sentinel-2, Landsat-8, crop mapping, urban agriculture region, phenology

## Abstract

Crop-type identification is very important in agricultural regions. Most researchers in this area have focused on exploring the ability of synthetic-aperture radar (SAR) sensors to identify crops. This paper uses multi-source (Sentinel-1, Sentinel-2, and Landsat-8) and multi-temporal data to identify crop types. The change detection method was used to analyze spectral and indices information in time series. Significant differences in crop growth status during the growing season were found. Then, three obviously differentiated time features were extracted. Three advanced machine learning algorithms (Support Vector Machine, Artificial Neural Network, and Random Forest, RF) were used to identify the crop types. The results showed that the detection of (Vertical-vertical) VV, (Vertical-horizontal) VH, and Cross Ratio (CR) changes was effective for identifying land cover. Moreover, the red-edge changes were obviously different according to crop growth periods. Sentinel-2 and Landsat-8 showed different normalized difference vegetation index (NDVI) changes also. By using single remote sensing data to classify crops, Sentinel-2 produced the highest overall accuracy (0.91) and Kappa coefficient (0.89). The combination of Sentinel-1, Sentinel-2, and Landsat-8 data provided the best overall accuracy (0.93) and Kappa coefficient (0.91). The RF method had the best performance in terms of identity classification. In addition, the indices feature dominated the classification results. The combination of phenological period information with multi-source remote sensing data can be used to explore a crop area and its status in the growing season. The results of crop classification can be used to analyze the density and distribution of crops. This study can also allow to determine crop growth status, improve crop yield estimation accuracy, and provide a basis for crop management.

## 1. Introduction

As a result of the development of the economy and the incessant extension of cities in China, the food security of major crop-producing areas (MCA) has become an important issue. One of the important methods for predicting agricultural productivity and ensuring food security is a timely inventory area of different crop types [1]. The classification and mapping of agricultural plants is extremely valuable for agricultural monitoring and food security [2].

Remote sensing technology based on optical or microwave sensors has become an important means of extracting crop planting areas, and one of the key technologies for remote sensing estimation is the identification of crop types [3]. Gradually, the classification results have been improved by increases in the spatial, temporal, and spectral resolution [4,5]. Large-scale spatial and time-series crop-type classifications have been the main study areas in this field [6,7,8]. However, the acquisition of optical images in key monitoring periods may be limited because of their vulnerability to rainy or cloud weather. A previous study showed that the acquisition of synthetic-aperture radar (SAR) data depends on the surface and subsurface characteristics (e.g., wavelength and roughness, geometry, and material contents) but is independent of solar illumination and cloud cover [9]. Furthermore, several reports found that multi-frequency SAR data have a greater influence on the classification performance than single-frequency SAR data [10,11,12]. However, SAR data are not used widely in agricultural areas because of their limited availability caused by, for example, poor-quality digital elevation models (DEM) and the more complex data structures required relative to optical data [9].

In recent years, the ever-increasing amount of high-spatial- and -temporal-resolution satellite data that have become available for free and the integration of multi-source images in time series have improved agricultural crop monitoring [13,14]. So far, some reports have shown that the integration of SAR and optical datasets can improve the performance of identification tasks in agricultural scenarios [15,16]. Kussul et al. [2] combined RADARSAT-2 and EO-1 to classify Ukrainian crops, a method that produced better accuracy. Skakun et al. [17] used multi-temporal C-Band Radarsat-2 Intensity and Landsat-8 Surface data to map crops in Ukraine and found that the combination of optical and SAR images can achieve better discrimination among crops. Erasmi et al. [18] used Landsat ETM+ and Envisat ASAR data to map land cover in Central Sulawesi, Indonesia. They found that the combination of textural information from SAR data with VNIR optical data improved the classification of vegetation. Zhou et al. [19] and Villa et al. [20] explored the potential to improve the accuracy of identification based on in-season crop mapping with the multi-temporal SAR and optical data, separately. Veloso et al. [21] used Sentinel-1 and Sentinel-2 data to map crop types and then estimated biophysical parameters. They found that the red edge wavelength has a higher sensitivity for the identification of vegetation information. Nicola et al. [22] used a fusion of Sentinel-1A and Sentinel-2A data for land-cover mapping in the lower Magdalena region, Colombia. Although these studies explored the potential to improve performance using SAR and optical data in crop or land-cover determination, the ability of spectral or indices features to identify crop areas and in-season growth has not been sufficiently examined.

So far, most studies have focused on using advanced sensors or on combining two types of sensor to monitor surface changes. A few studies have used a combination of more than two sensors to study crop-type identification using features of change detection in a time series. Some scientists have studied the forest type, the leaf area index (LAI) of vegetation, and so on. Liu et al. [23] used Sentinel-1A, Sentinel-2A, multi-temporal Landsat-8, and DEM data with Random Forest (RF) to identify forest types. The results showed that the synthesis of SAR, optical, and multi-spectral images can produce satisfactory results. Gray and Song [24] mapped the LAI by combining spectral information from Landsat, spatial information from IKONOS, and temporal information from MODIS. The results showed that the use of multi-dimensional information from multi-source remote sensing features (spectral, spatial, and temporal information) improved LAI mapping significantly. Zhou et al. [25] selected multiple feature dual-polarimetric Sentinel-1A, Landsat-8 OLI, and Hyperion EO-1 images to classify urban land cover, which produced higher classification accuracy. Torbick et al. (2017, 2018) tried to monitor rice agriculture with a time series of Sentinel-1 data combined with Landsat-8 and PALSAR-2 data in Myanmar and synergized Landsat-8, Sentinel-1, and Sentinel-2 data to explore the ability to identify crop types in the USA [9,26]. The results showed that the combination of data from multiple sensors can achieve a high level of accuracy. However, this method was only used in simple agricultural regions and cannot be employed for composite agricultural and urban regions.

Several studies have explored the performance of different classification algorithms in various fields [9,27,28,29,30]. In recent years, the Support Vector Machine (SVM), RF, and Artificial Neural Network (ANN) have become very popular classifiers for SAR data or optical data classification, as they can provide accurate land cover maps [16,24,31,32]. Torbick et al. [9] used the RF algorithm to map land use and land cover classification by fusing Sentinel-1, Landsat-8 OLI, and PALSAR-2 data. Graves et al. [33] used the Support Vector Machine (SVM) model to discriminate among tree species and made abundance predictions in a tropical agricultural landscape. ANNs are commonly used for remote sensing image discrimination and often achieve the same level of accuracy as RF and SVM [34]. However, few reports have studied the potential for ANN, SVM, and RF classifiers to map crops. It is necessary to compare the ability of these three algorithms to map crops.

This study aimed to quantify the potential of three different sensors (Sentinel-1 backscatter, optical Sentinel-2, and Landsat-8) to monitor crop dynamics during the growing season. The features derived from SAR and optical data related to different crop types changed at different stages within the growing season. To determine the identification performance of synergized Sentinel-1, Sentinel-2, and Landsat-8, spectral features, index information and texture features were derived from the multi-source remote sensing data. A comparison of three advanced machine learning algorithms was completed to produce an optimal classification model for mapping crops. This study is particularly important as it contributes to the improvement of crop-type mapping with multi-sensors and multi-temporal data in urban agriculture scenarios. This study provides effective data on crop planting adjustment and yield precision in urban agricultural regions.

## 2. Study Area and Materials

### 2.1. Study Area

**Figure 1:**
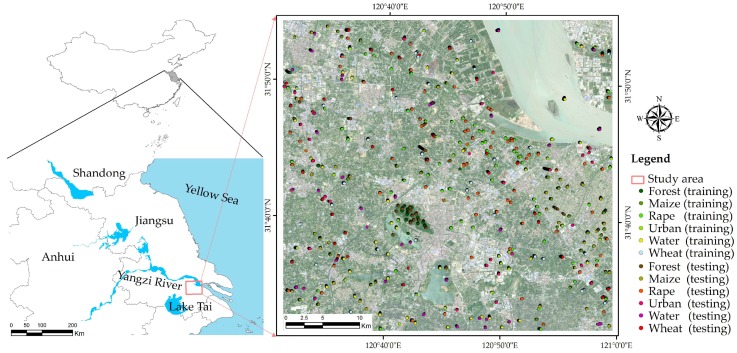
The study area based on the Sentinel-2 multispectral image (Red, Green, Blue). The location of training samples and testing samples is shown in the image.

The study area is one of the main crop product regions and is located at the lower reaches of the Yangzi River in China (120°33′–121°03′ E, 31°31′–31°50′ N), covering an area of over 1800 km^2^ (Figure 1). This region has a subtropical monsoon climate with an average annual temperature of 16.2 °C and an average annual precipitation of 1076 mm. The average annual number of sunshine hours in this region is 1813.4 h, and the sunshine percentage is 41%. The dominate terrain is plains, and the land slopes from northwest to southeast. The study area is an MCA in the south of China based on the excellent topography and climatic conditions. In the MCA, the climate is classified as a subtropical monsoon climate. The major crops in the area include rice, wheat, rapeseed, maize, beans, and tea. There are two crop growth periods per year. The wheat and rapeseed planting season usually occurs in the winter season from late November to June, and the maize planting season ranges from March to June (Table 1). In 2017, the sown area of various crops in the city was 44,730 ha, with a yield of 2,611,000 T (2017 Changshu yearbook).

### 2.2. Data Acquisition and Preprocessing

#### 2.2.1. Satellite Dataset

Sentinel-1A (S1) images were acquired from the Wide Swath mode (IW) Ground Range Detected in High Resolution (GRDH) from the European Space Agency (ESA) Sentinels Scientific Data Hub. The Sentinel 1A satellite is a dual polarization (VV+VH) C band (5.36 GHz) SAR data product. In the study, 14 Sentinel-1A images were acquired from December 2017 to May 2018. EAS SNAP software was used to process images, which included filtering, radiometric calibration corrects, and geocoding of terrain correction. The inherent speckle noise was filtered using the filter method with a 5 × 5 window size. All images were geocoded using the Doppler method with a spectral resolution of 5 m × 20 m and a temporal resolution of 12 days. This was a necessary calibration process which was done with the mosaicking of discrete data. Detailed information of the 14 Sentinel-1A images is shown in Table 2.

Sentinel-2A/B (S2) images were acquired with a multispectral instrument (MSI) from ESA, which included 13 bands with different resolutions, including 4 bands of 10 m resolution—band 2 (490 nm), band 3 (560 nm), band 4 (665 nm), and band 8 (842 nm)—6 bands of 20 m resolution—infrared wavelength band 5 (705 nm), band 6 (740 nm), band 7 (783 nm), band 8a (865 nm), band 11 (1610 nm), and band 12 (2190 nm)—and 3 bands of 60 m resolution—band 1 (443 nm) band 9 (946 nm), and band 10 (1374 nm). The studied wavelengths incorporate two spectral bands in the so-called red-edge region, which are sensitive to vegetation, which is important for the retrieval of chlorophyll content [35,36]. S2 data were radio-metrically calibrated and atmospherically corrected using the ESA’s Sen2Cor algorithm in SNAP version 5.0.8 [37]. The 10 types of acquired data covered the winter crop growing season from December 2017 to May 2018 (Table 2).

The Landsat-8 (L) image was produced by the Operational Land Imager (OLI) and Thermal Infrared Sensor (TIRS). The image carried nine spectral bands, of which eight bands had a spatial resolution of 30 m (coastal: 443 nm, blue: 485 nm, green: 563 nm, red: 655 nm, NIR: 865 nm, SWIR1: 1610 nm, SWIR2: 2200 nm, and cirrus: 1375 nm) and one panchromatic band had a spatial resolution of 15 m. Radiometric calibration and atmospheric correction were performed to remove the various sources of error. Five Landsat-8 images were acquired which were produced with UTM/WGS84 projection (Table 2).

#### 2.2.2. Field Sample Data

The study combined GNSS (Global Navigation Satellite System) field surveys and reference Google Earth images. Eight classes of surface type were identified: forest, maize, rape, urban, water, and wheat. We chose samples by visual interpretation of field validation and Google Earth images (image date: 6 May 2018). Then, the ground sample points were randomly separated into parts (50% training and 50% testing), and the accuracy was calculated (Table 3). The location of training samples and testing samples is shown in Figure 1.

### 2.3. Crop Classification Method

#### 2.3.1. Features Described for Crop Classification

##### Spectral Information

A previous study showed that the use of spectral information to extract the mean, standard deviation, and variance of each band can separate the different features of crop types [38]. Here, with the object-based method, we derived the spectral features using all bands of the three types of remote sensing data to calculate the mean, standard deviation, and variance, separately [39,40]. For the SAR dataset, the incidence angles of the data were approximately 40° (38° to 41°), which was suitable to identify the crop parameters [22]. Both (vertical-horizontal) VH and (vertical-vertical) VV values can contain vegetation–ground interactions. The spectral information came from the polarization of the VV band and VH band and the Cross Ratio (CR) between the VH and VV bands in the Sentinel-1 dataset. The CR is the ratio of the VH and VV mean values, which can reduce the double-bounce effect, systematic errors, and environmental factors, thereby bringing out more useful information [3]. The spectral features of optical data are often extracted for the identification of surface information. For the Sentinel-2 and Landsat-8 datasets, the mean, standard deviation, and variance were calculated as the spectral features in each band. Table 4 shows the details of the spectral features.

##### Indices Features

The use of red-edge bands from Sentinel-2 data to characterize vegetation has been shown through simulation studies [45,46]. Therefore, we derived the features based on the NIR narrow band (band 8A) and red-edge band information. Some research has argued for the potential of vegetation indices in the classification of agricultural regions [22,36,47]. This study also used multi-resource optical images where the indices features extracted from Sentinel-2 images and Landsat-8 data were consistent with the vegetation indices, such as the Enhanced Vegetation Index (EVI) [41], Triangular Vegetation Index (TVI) [42], Normalized Difference Water Index (NDWI) [43], and the Normalized Difference Tillage Index (NDTI) [44]. The TVI and EVI can be used to estimate the green leaf area index and canopy chlorophyll density. NDWI and NDTI reflect the biochemical metrics of crops. Thus, we selected 11 indices of Sentinel-2 data and 5 indices of Landsat-8 data to identify crop types. The details of indices features are shown in Table 4.

##### Textural Features

Textural information relates to the structural features of the target surface and the surrounding environment, which can also reflect spatial variation in land cover. As such, the textural features can be extracted by statistical, structural, and spectral methods [48]. The potential for textural features from satellite images to be used in the identification of crops has been demonstrated to be significant [20,48]. Here, a texture analysis method based on the Gray-Level Co-occurrence Matrix (GLCM) was adopted. Eight variables based on SAR and multispectral data were calculated, such as the mean (ME), variance (VA), homogeneity (HO), contrast (CON), dissimilarity (DI), entropy (EN), second moment (SM), and correlation (COR). In addition, a principal component analysis (PCA) was carried out to achieve more accurate classification results based on the reduction of high-dimensional and redundant texture features. A textural analysis was carried out, and the size of the window was 3 × 3. This study extracted the textural feature from the Sentinel-1, Sentinel-2, and Landsat-8 datasets separately (Table 4).

The spectral indices and texture features are relevant to phenotyping. The texture information shows the crop density and shape features. The indices (e.g., NDVI) information shows the crop growth status in a growth period. The spectral information of red-edge bands in Sentinel-2 data shows a potential performance application in evaluating the growth status of crops. The optical data (Sentinel-2 and Landsat-8) monitor color changes in the crops during the growth period.

#### 2.3.2. Statistical Analysis and Classification Modeling

Using the crop class samples, the changing characteristics of crops in the growing season based on spectral features, indices, and textural features were detected (Table 5). In the study, we used a total of 73 features in each single dataset and combined 7 combinations of data variables to establish the following crop classifications: S1 (textural features, spectral features), S2 L (textural features, spectral features, and indices), S1+S2, S1+L, S2+L, S1+S2+L. Table 4 shows the variables derived from multi-sensor images, which included spectral features, textural features, and indices from S2 and L, and spectral features and textural features from the S1 backscatter coefficient in time-series images, to determine the best configuration for crop classification.

#### 2.3.3. Classification and Assessment Accuracy

The extracted features were employed to identify crop classifications by three advanced machine learning/classification methods, namely, SVM, ANN, and RF.

The SVM algorithm tries to maximize the margin between classes (the crops) through finding the optimal hyperplane in the n-dimensional classification space [49]. We used the SVM classifier with a radial basis function (RBF) kernel using LIBSVM [49]. ANN simulates the human brain nervous system recognition structure with a high degree of non-high linear classification capacity [50,51]. The multi-layered perceptron is a commonly used type of neural network. This type of ANN generally consists of three or more layers that can separate nonlinear data [52,53]. The RF classifier is commonly described as an ensemble of decision trees where class labeling is achieved by voting. It can handle high-dimensional data and is relatively resistant to overfitting [54]. RF also determines the importance of features (texture, spectral, and indices features) in the classification process [55,56]. We used Python statistical software along with Scikit-learn package to implement these classification techniques [57]. Further, 13 feature scenarios were tested with the machine learning methods. The classification accuracy is reported for each scenario, and the classification results were compared on the basis of their accuracy in crop mapping for each crop class, as described in Section 3.3.2.

Finally, we calculated a confusion matrix for each classification result based on our ground control points. Then, the overall accuracy (OA), Kappa coefficient, producer’s accuracy (*P*), and user’s accuracy (*U*) were calculated to evaluate the classification results [58,59]. The *F*1 measure (Equation (1)) was calculated to evaluate the effectiveness of the crop classification [60]. The *F*1 accuracy is considered to be more meaningful than the Kappa coefficient and the overall accuracy. The value range of *F*1 is from 0 to 1—the larger the *F*1 score is, the more accurate the classification results are. The *F*1 score is the harmonic mean of *U* and *P*:

(1) $$F1\;=2\times \;\frac{P\times U}{U\;+\;P}.$$

An additional parameter for image classification accuracy is the Figure of Merit (*FoM*) [61,62,63]. The *FoM* computes from omission, commission, and overall agreement (Equation (2)):

(2) $$FoM\;=\frac{a}{o+a+c}\times 100\%$$

In the equation, *a* represents overall agreement, *o* represents overall omission numbers, *c* represents overall commission numbers.

## 3. Results

### 3.1. Deriving Features to Identify Crops in Time Series

#### 3.1.1. Effectiveness of VH, VV, and CR Features Using Sentinel-1 Data

**Figure 2:**
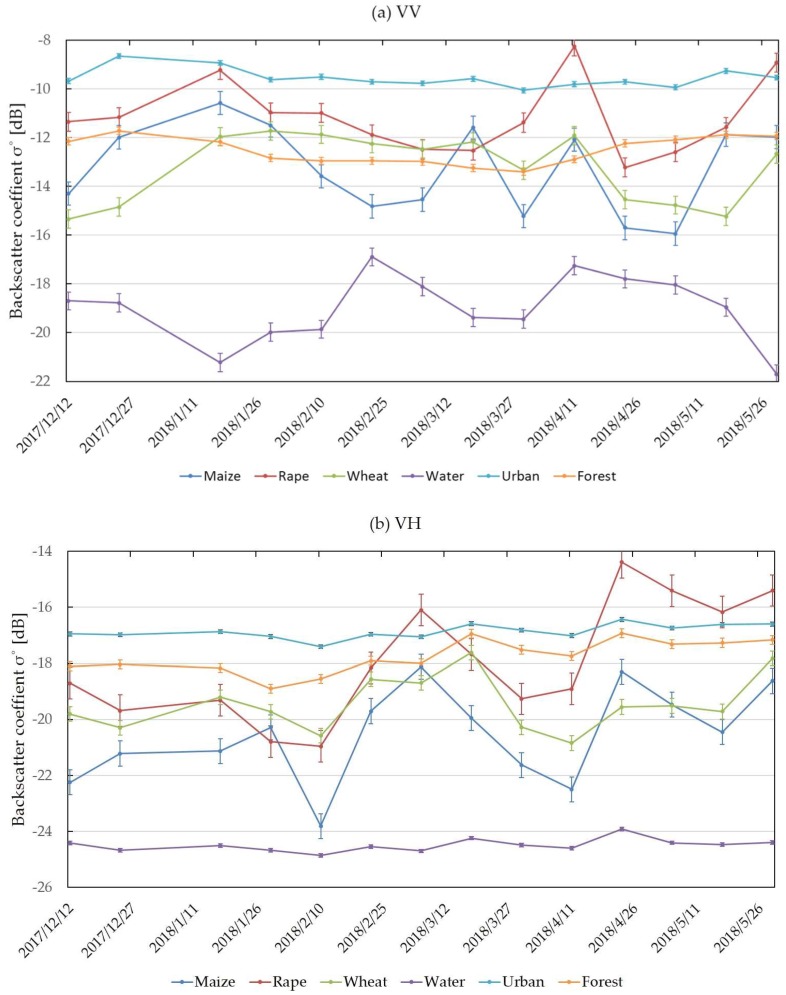

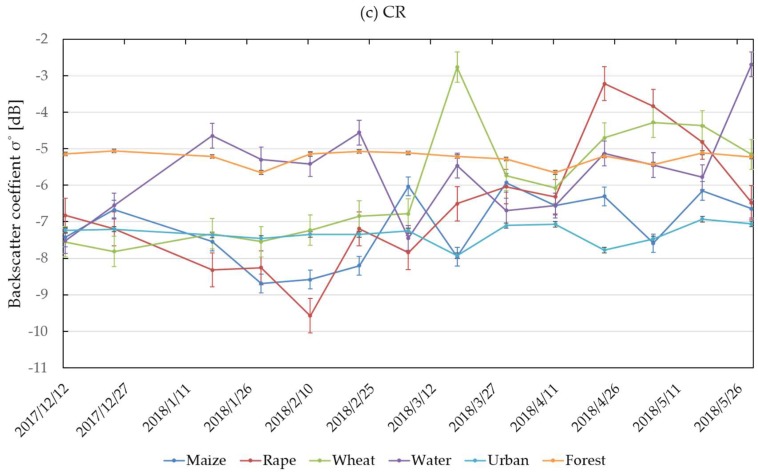
Six land cover features of Sentinel-1 backscatter coefficients of a time series: (**a**) (vertical-vertical) VV, (**b**) (vertical-horizontal) VH and (**c**) Cross Ratio (CR).

The study used training sample data which covered a complete growing season (from December 2017 to May 2018) of three crops using Sentinel-1 polarization data to monitoring the crop growth changes. Figure 2a–c shows the backscatter coefficient changes of the five land-cover classification time series. It can be observed that the mean VV backscatter coefficient values (Figure 2a) of maize, rape, wheat, urban, water, and forest were −13.26, −11.18, −13.22, −19.01, −9.55, and −12.53 dB, respectively. The mean VH backscatter coefficient values (Figure 2b) were 20.53, −17.92, −19.44, −24.49, −16.86, and −17.75 dB, respectively. The mean CR values (Figure 2c) were −7.16, −6.60, −6.01, −5.65, −7.32, and −5.25 dB, respectively. In addition, the VV and VH values of urban and water were relatively independent and separable for most of the growing period. The results showed that it was feasible to distinguish crops from other land-cover types using VV and VH features.

#### 3.1.2. The NDVI Characterized Crops in Time Series of Sentinel-2 and Landsat-8 Data

**Figure 3:**
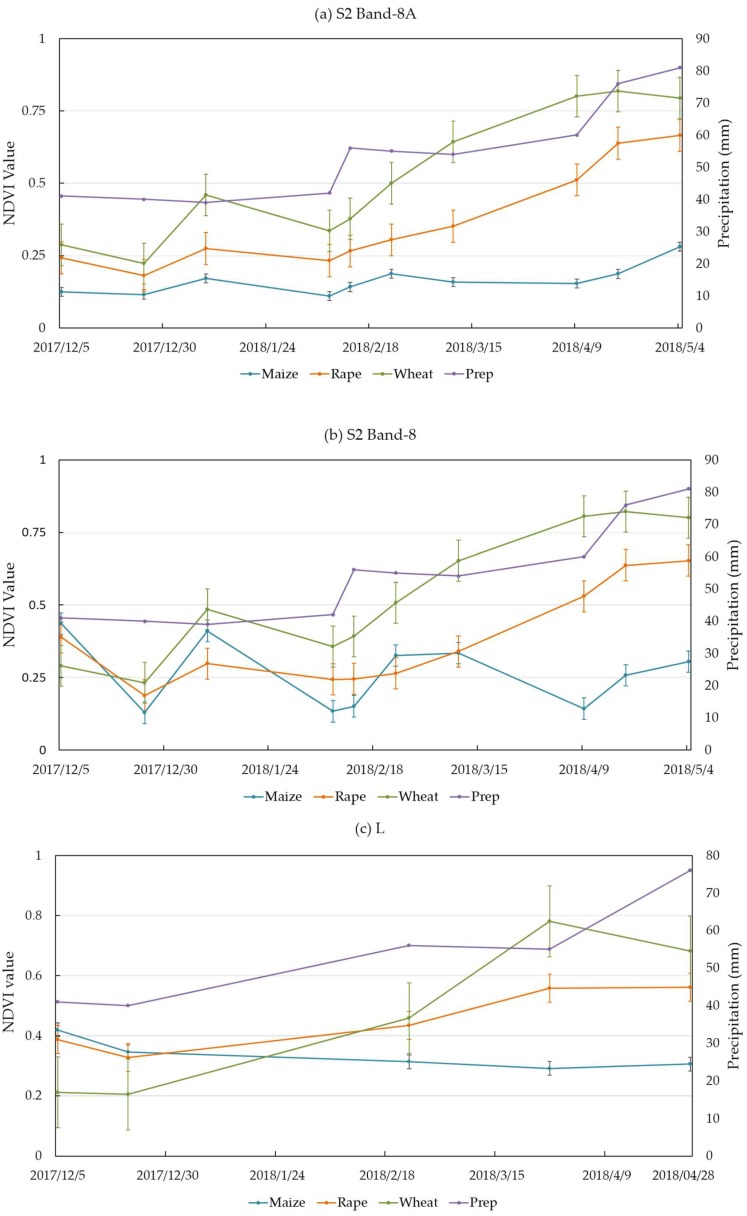
The NDVI band plot shows that all the three crops had obviously differentiated in the growth season.

Figure 3a–c compares the NDVI values of S2 Band-8A, S2 Band-8, and L and shows that the NDVI increased with the precipitations. Figure 3a shows that rapeseed and wheat had similar NDVI values in the early growth season. Moreover, they significantly differed in similar seasons. Because the phenology and structure of the two crops are obviously different, they had significant differences in the intermediate and late seasons. Maize did not significantly change from the early season to the grown season. Moreover, the total tendency of NDVI change was an increase when there was a large amount of precipitation. Precipitation in the growing season increased from December 2017 to May 2018. In Figure 3b, the three crops are shown to be different throughout the whole growth period. Wheat and rapeseed showed larger increases from December 2017 to May 2018 than maize. This is because the maize growth period began in March 2018. During growth, all of them showed more differences. Figure 3c L shows that the three crops had similar NDVI values during the growth period. It is obvious that there were large differences in the middle growth period. When comparing S2 Band-8A (Figure 3a), S2 Band-8 (Figure 3b), and L (Figure 3c), the S2 Band-8A showed more differences over the whole growth period. This certificated that the NDVI edge is more sensitive than the NDVI spectral features. This result could provide an effective way to identify the status of crop growth.

#### 3.1.3. Indices Features in Sentinel-2

**Figure 4:**
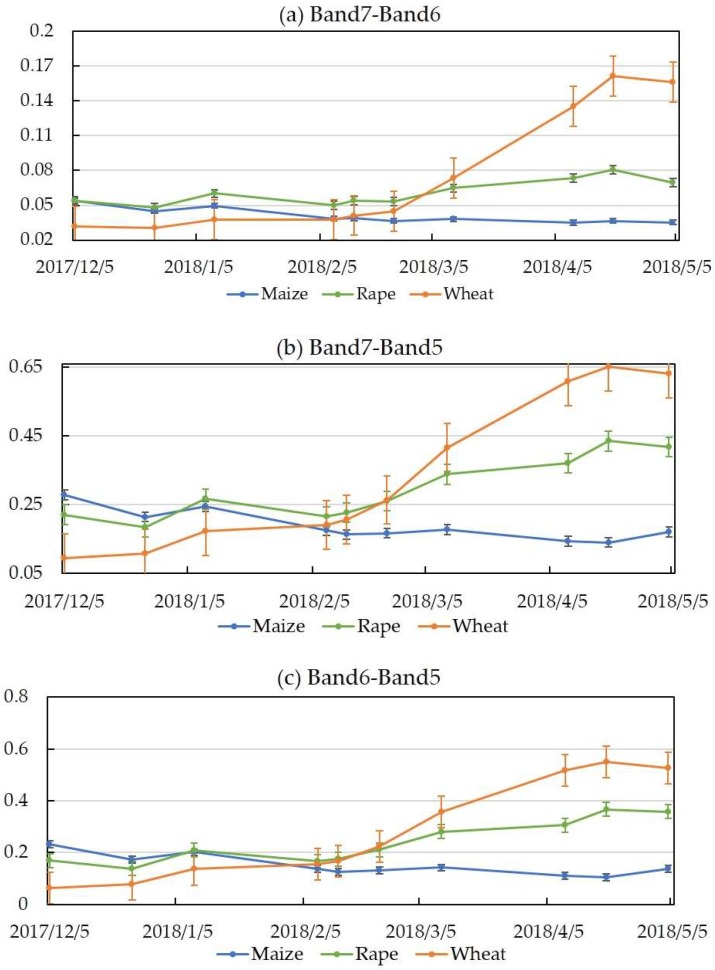

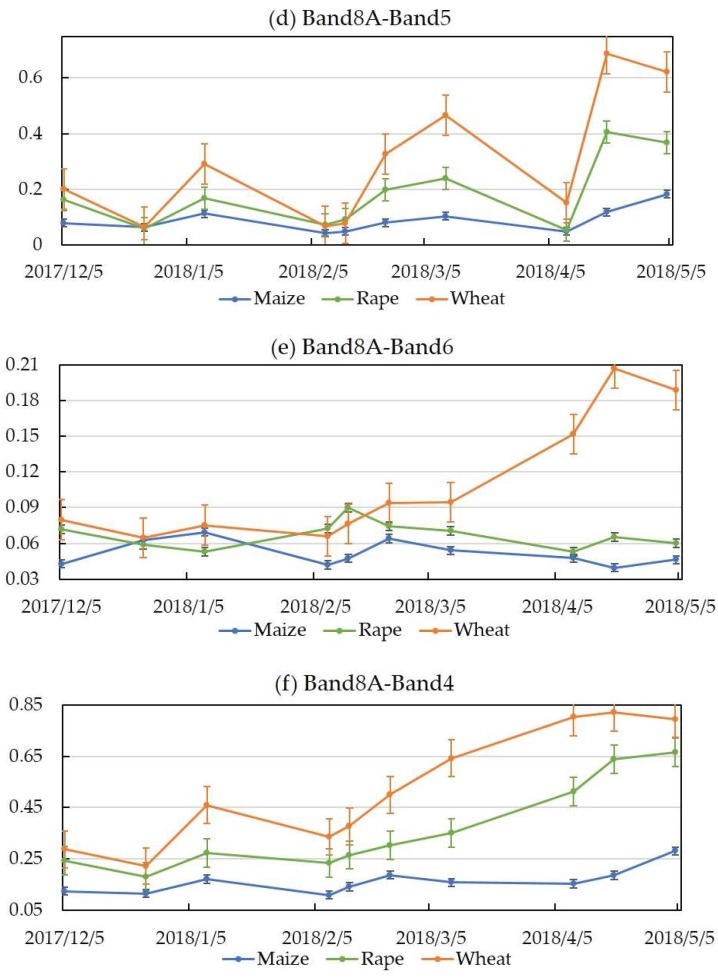
Time-series changes at in red-edge bands of Sentinel-2 data.

Figure 4 shows the results of the comparison of indices derived from the red-edge bands of S2. The results show that the three crops changed during the time series (Figure 4) acquired in the four red-edge bands with S2. Most of the indices showed a similar increasing tendency in the growth season. Figure 4a–f shows obvious independent and separate indices in March, April, and May. At the same time, Figure 4a–d shows similar indices in February.

Obvious increases in wheat were observed during the growing season for all indices (Figure 4). The maximum values of wheat and rape appeared in May 2018. In addition, there were obvious differences for each crop. Maize indices differed in value weekly during the growing season. Notably, the three red-edge and NIR bands in the January, March, and May images showed high separation between the crop species. As such, we selected the data combination of January, March, and May for each year for crop-type classification.

**Figure 5:**
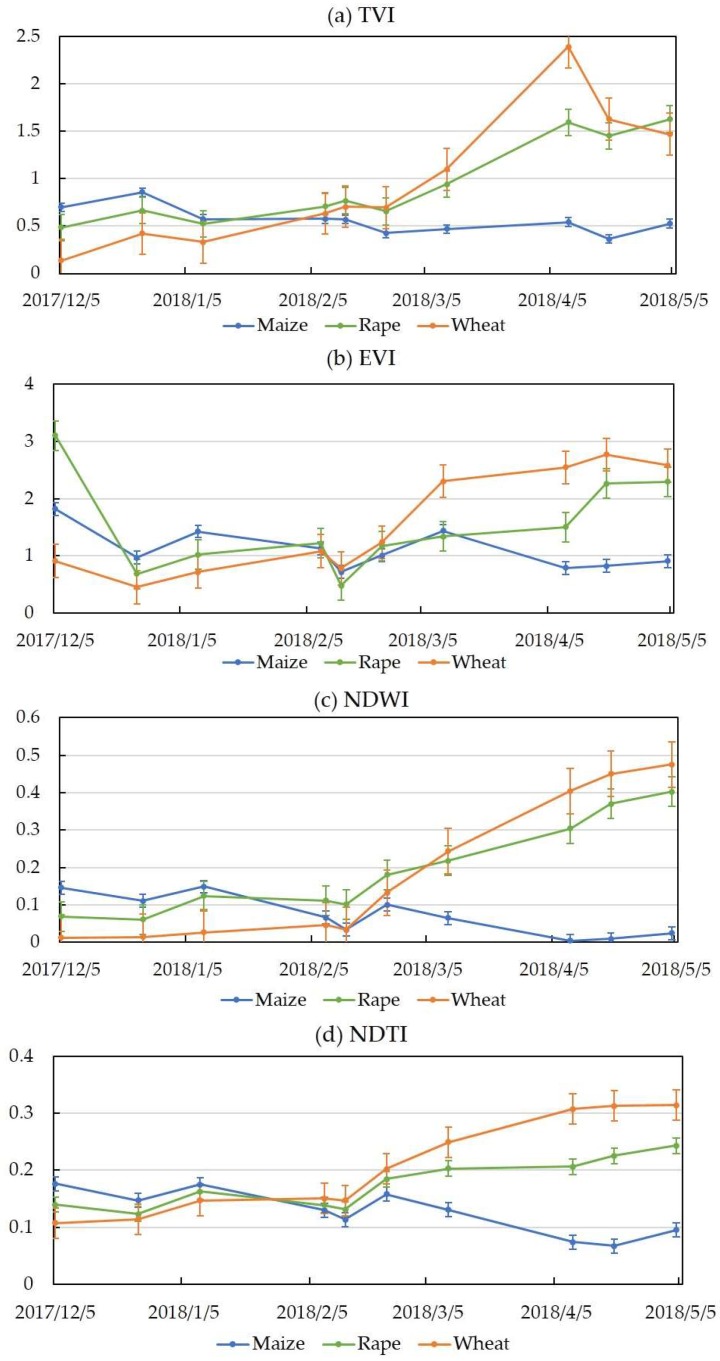
Indices of maize, rape, and wheat in time series for Sentinel-2 data.

Figure 5 shows the time series of the TVI, EVI, NDWI, and NDTI indices of the three crops (maize, rapeseed, and wheat). The results indicated that most of the indices could be separated from March to May and they were especially significantly independent during the maturity period of crops. All the indices were better separated in the late grow season than in the early grow season. This can be explained by the fact that the leaf water content of wheat, rape, and maize increased differently as their grow status changed. Moreover, maize showed obviously different changes compared with wheat and rape from the March to May. This is because maize has a different growing season (from March to June) and phenological period compared with wheat and rape. In summary, the indices of optical images showed a potential to identify crop types.

### 3.2. Assessment Accuracy

#### 3.2.1. Comparison of Features Extracted with the Different Sensors

To evaluate the performance of different sensors and different features, S1, S2, and L were used to classify crop types with RF, SVM, and ANN methods separately. Table 6 shows that the highest accuracy was acquired (which was highlighted) using three features (textural and spectral features and indices) from three datasets (S1, S2, and L) separately. This means that the total information from the three features was used to acquire better accuracy than would have been achieved with just one or two features. For S1, the textural features of Sentinel-1 data showed better overall accuracy than the spectral features. This is because the textural value of the SAR backscatter coefficient differed obviously with land cover, as it depends on shape and roughness; this result is similar to the results of Veloso et al. and Jia et al. [11,21]. When textural and spectral features were combined, the overall accuracy increased. Using the features derived from the S2 data, the maximum accuracy was 0.91 with the RF classifier. Meanwhile, the L data also had a maximum accuracy of 0.86, which was derived using textural, spectral, and index features with the RF classifier. It was clearly shown that by using S2 features for classification, it is possible to achieve better overall accuracy than by using the other data. Further, the combination of three features of each type of data increased the overall accuracy.

**Figure 6:**
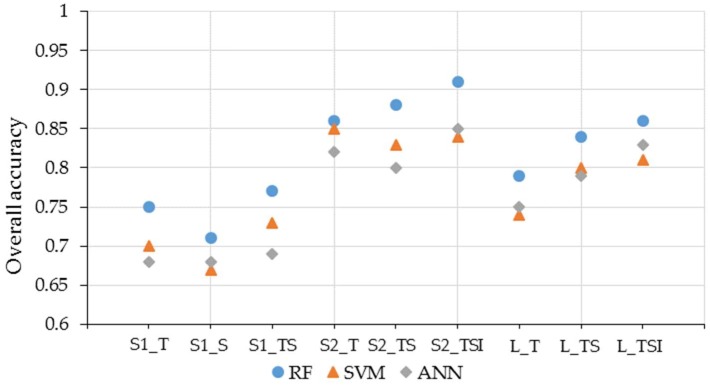
Performance of the three classifiers for nine classification scenarios.

Figure 6 compares the effects of the RF, SVM, and ANN classification algorithms. All scenarios showed that the RF classifier performed better than the SVM and ANN classifiers. The SVM and ANN classifiers produced similar classification results. When using single datasets, the classification accuracy of SVM was slightly higher than that of ANN. Then, the number of features was higher than when using a single dataset. When the three data types were combined, the accuracy of ANN was higher than that of SVM; conversely, RF’s accuracy remained the best.

#### 3.2.2. Accuracy Assessment of Combined SAR and Optical Data

A further accuracy assessment for the classification of combined data was performed on the basis of OA, Kappa, and F1 and *FoM* values. Four classification scenarios were compared to achieve the optimal combination of multi-source datasets. The classification schemes are presented in Table 7, where the remote sensing imagery, OA, Kappa coefficient, *F*1, and *FoM* are listed for each tested scenario. Table 7 shows that the best model composition was the combination of S1, S2, and L data with the RF classifier, which gave an OA value of 0.93 (which was highlighted). According to the comparison, the values resulting from the combinations were higher than those obtained using just S1 data. Compared with single optical data, the combinations of S1 with L and S2 with L did not significantly increase the overall accuracy. The combinations of S1 with S2 and of S1 with S2 and L gave better overall accuracy. This showed that L weakly affected the classification result.

**Figure 7:**
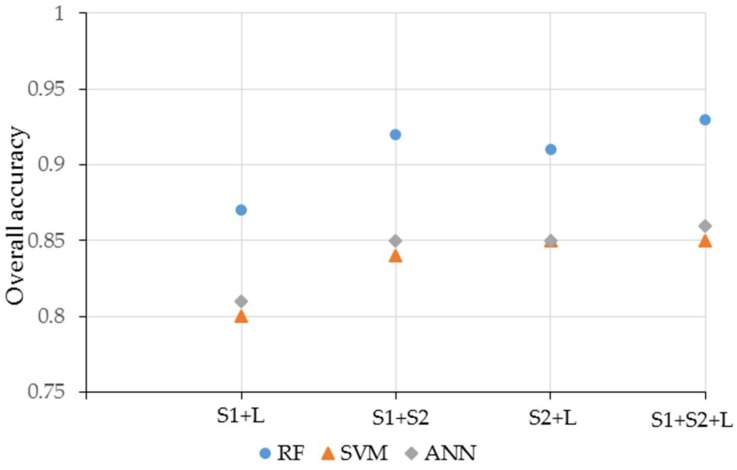
Performance of the three classifiers from Figure 6 for the four classification scenarios.

Figure 7 summarizes the performance of the three different classifiers using four classification scenarios. RF obviously provided better classification results than the SVM and ANN classifiers. The classification performance often affects not only the number of feature but also the relationships among variables. The results of each classification showed that it was easy to discriminate between wheat and water (Table 7).

### 3.3. Crop Mapping Using the Optimal Combination

#### 3.3.1. Optimal Classification Combination for Crop Mapping

According to the comparison of the combinations of the three types of data, the best vector combination was S1, S2, and L with the RF classifier. Table 8 shows the confusion matrix results of the optimal classification combination, including *U*, *P*, *F*1, and *FoM* values for the classification of six classes. It shows that *U* had better accuracy for maize, water, and wheat, and *P* was better for water and wheat. The results further show that the maximum *P* and *U* were for water and wheat. Then, the majority of errors were for forest, rape, and wheat. This could be due to the fact that in the transition area of forest, wheat, and rape, the optical data were dominant over similar spectral information. In additional, rape and urban also showed error classes. It is known that greenhouse planting is used in the early growing season in the study regions. The accuracy of the classification results is affected when using the traditional identification method.

**Figure 8:**
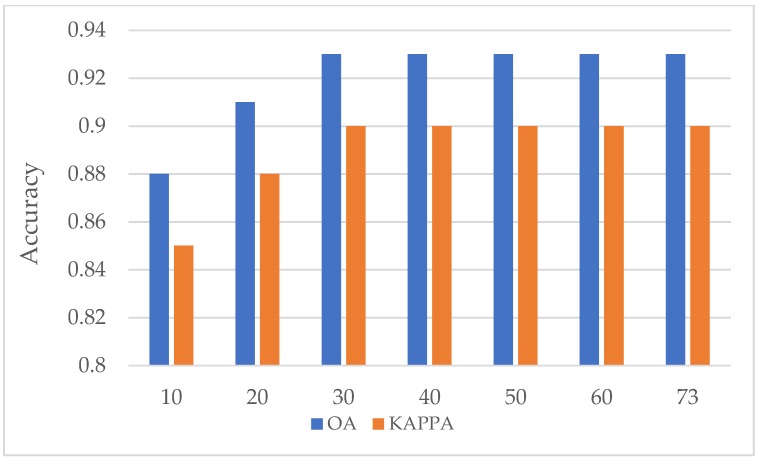
Accuracy results with different variable numbers.

**Figure 9:**
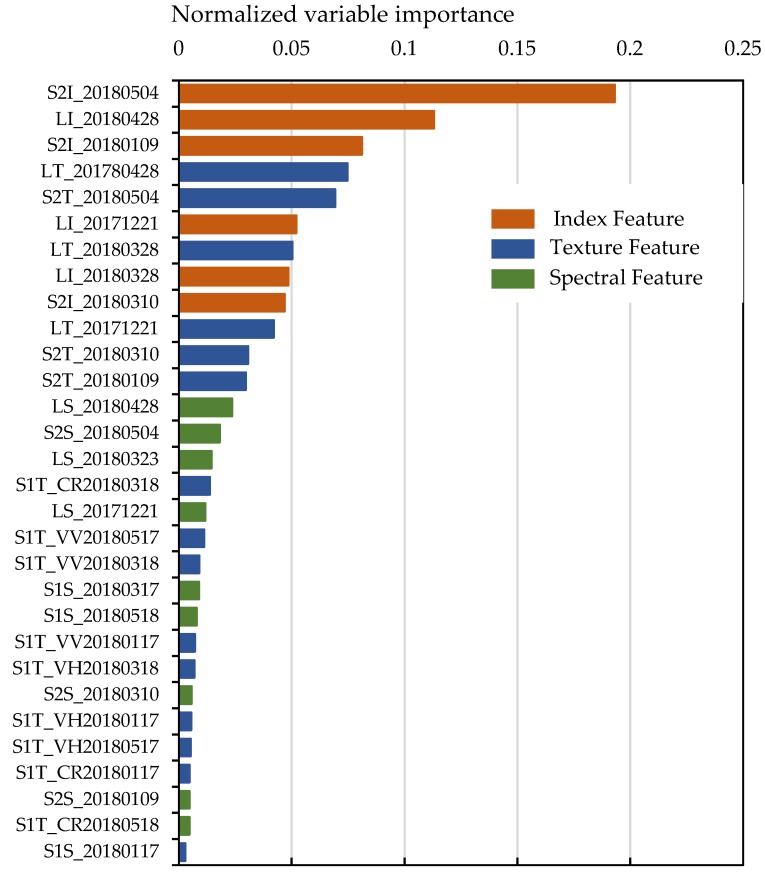
Importance of variables in the RF classification process.

The RF classifier also ranked the features according to their importance, which could provide some useful information to improve the classification process. Figure 8 shows the variable importance of features derived from S1, S2, and L data. The index features were shown to be dominant in the classification result. Meanwhile, Figure 9 shows that the index features derived from S2 and L data separately played more important roles than the other features. Figure 9 shows that in May, the indices in S2 dominated the classification results. The crops grown in the late season had a more important effect on crop classification.

#### 3.3.2. Mapping Crop Types and Land Cover

**Figure 10:**
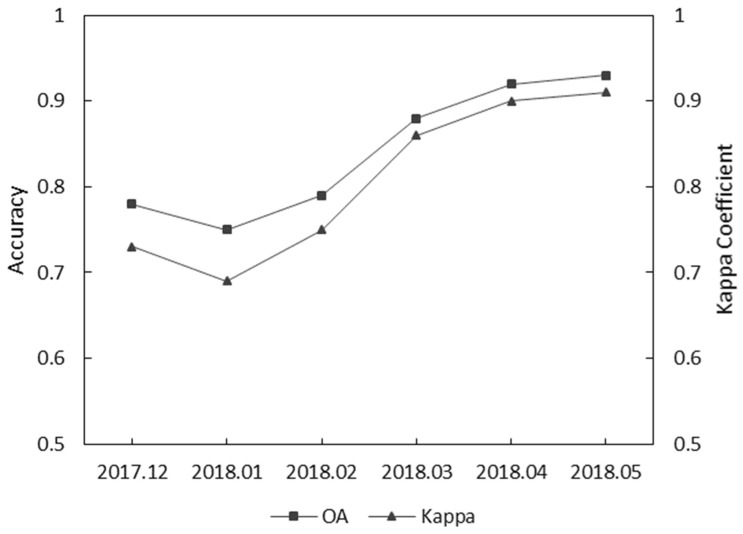
OA and Kappa coefficient for the combined S1, S2, and L data in the time series.

Figure 10 shows the performance of the combination of S1, S2, and L data to identify crops in the crop growing period. It is obviously shown that in the flowering period (May), the crops acquired the best OA and Kappa coefficient. The plot shows a tendency for the accuracy to decrease in January 2018, which is because the rapeseed and wheat were covered by snow or rain in the winter season. After greening (February 2018), rape and wheat leaf can, obviously, be monitored. During March 2018, the accuracy increased until it reached its highest value; the reason for this is that maize was sowed, and the crops flowered (April 2018) and matured (May 2018).

**Figure 11:**
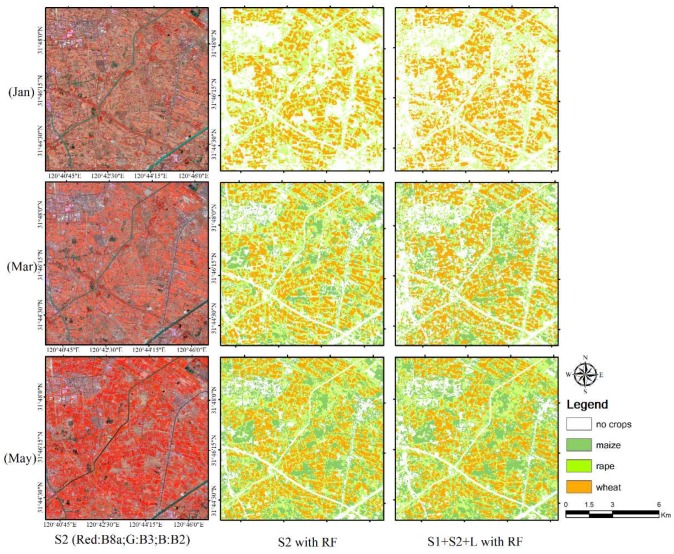
The best classification results of single and multi-resource data in January, March, and May 2018.

Figure 11 shows the S2 satellite data and crop mapping in January, March, and May with S2 features and the combination of S1, S2, and L features separately. It clearly characterizes the changes of crop distribution in different phenological periods.

## 4. Discussion

It is known that the SAR sensor contributes to identify earth surface information. This was confirmed in this study. The CR value of the total land cover was observed to vary for the majority of the time. Moreover, the three crops were obviously independent and separable from the heading to the maturing stage in terms of phenology. The results indicated that the CR could effectively separate the three crops in the late period of crop growth. Mattia et al. and Satalino et al. also showed that phenology and vegetation structure changes most likely dominate the change in CR during stem extension and heading [21,64,65]. In addition, both the decrease and the strong increase related to phenology (flowering and ripening) also contribute to this change, as also observed by Wiseman et al. [66] and Veloso et al. [21]. This indicates that CR is sensitive to the crop growing status.

For the optical sensor, Sentinel-2 appeared more sensitive for retrieving crop information than Landsat, especially in the near-infrared spectral range. This is due to its narrow band. We also took advantage of the fact that Sentinel-2 is equipped with red-edge bands in the 4–7 channels. This spectral range is significantly sensitive, therefore useful, for monitoring vegetation changes. Some literature has indicated that vegetation indices are among the oldest and most widely used tools to estimate LAI and chlorophyll [45,67]. Delegido et al. confirmed that the red-edge bands are sensitive for LAI and chlorophyll measurements [46]. They can be used to infer the growth status of crops in the growing season. Figure 4 shows the obvious changes of three different crops in the growth season. In fact, we took advantage of these characteristics to separate the crop types in time series.

The results showed that SAR data (S1) clearly identified different types of land cover but performed weakly in the identification of similar crop types, such as maize and rapeseed. This is in accordance with the results of Section 3.1.1. Although S2 and L could be effectively used to identify different crops, S2 showed better accuracy in crop-type identification than L and S1, especially when combined indices were used. This is because the edge bands in S2 have a strong sensitivity for vegetation [68]. We used *F*1 score and *FoM* to assess the classification accuracy. Previous studies showed that F1 plays an important role in the assessment of classification performance [19,25,69]. We also found that *FoM* can assess classification performance and, in fact, was used in previous researches [61,62,63]. We found these two metrics performed similarly in assessing classification accuracy. It should be noted that our final optimal classification for crop map was obtained from a combination based on *F*1 and *FoM* measurements; in other words, the map with optimal classification presented the highest *F*1 and *FoM* values for each crop type in our study area. We used *F*1 and *FoM* to certify that they were more robust and reliable than *U* or *P* [58,61,69].

The RF classifier produced the best overall accuracies for mapping both whole forests (OA and kappa coefficient) or forests with a certain crop type (*F*1 and *FoM*), whatever independent feature or combined features were used for classification (using single S1, S2, L features, or combined S1 and S2, or S1, S2, and L features). Our crop map was based on the RF classification results using combined features of S1, S2, and L. In fact, the RF classifier provided an assessment of variable importance, which showed the dominant variables of the classification results (Figure 9). So, we obtained the optimal accuracy in the maturing period according to the phenology of the crops (Figure 10). The use of the edge band obviously contributed to an increase in the crop classification accuracy because of its sensitivity to the vegetation information. This result is similar to that found in references [21,22,40]. At the same time, the L dataset did not dominate the classification result but could increase the time resolution in time-series crop classification (Table 8). The index derived from S2 is useful for crop classification.

## 5. Conclusions

This paper explored the potential for the use of multi-resource remote sensing data to identify crops of the urban agricultural region. We derived three feature types (spectral, textural, and index) which were proven to affect different crops in the growing season. Then, using three advanced machine learning methods, we selected an optimal mapping method based on their *F*1 and *FoM* accuracies and derived the final land-cover map. The findings in this study include the following:

(1) The use of Sentinel-1 data affected the land-cover classification. However, their ability to identify crop type was weaker than that of optical data. The red-edge band of Sentinel-2 was more sensitive than the normal band of L to vegetation information. The single use of the Sentinel-2 showed higher accuracy than the use of Sentinel-1 or Landsat-08 data.
(2) The Random Forest classifier generally produced highest performance in terms of overall accuracy (OA), Kappa coefficient, and *F*1 values for mapping crop types for any classification scenario.
(3) The use of the combination of Sentinel-1, Sentinel-2, and Landsat-08 in the time series provided an optimal crop and land cover classification result. The assessment of the importance of the RF variables also showed that in May, index features dominated the classification results.

## Figures and Tables

**Table 1:** Dates (month. day) of winter wheat, rape, and maize phenological stages, as recorded at Changshu station from November 2017 to June 2018.

Phenology		Sowing	Seedling	Tillering	Over-Wintering	Greening up	Jointing	Booting	Heading	Flowering	Maturing
**Data**	**Wheat**	11.15	12.05	12.20	1.30	2.26	3.10	4.05	5.01	5.06	6.08
	**Rape**	10.25	11.15	─	─	─	─	2.25	─	4.15	5.20
	**Maize**	3.05	3.25	─	─	─	─	4.10	5.10	5.25	6.10

**Table 2:** Description of the parameters of Sentinel-1 products.

Dataset	Date	Resolution	Source
14 Sentinel-1	12/12/17, 12/24/17, 01/17/18, 01/29/18, 02/10/18, 02/22/18, 03/06/18, 03/18/18, 03/30/18, 04/11/18, 04/23/18, 05/05/18, 05/17/18, 05/29/18	5 × 20 m	(EAS, 2017)
10 Sentinel-2	12/05/17, 12/25/17, 01/09/18, 02/08/18, 02/13/18, 02/23/18, 03/10/18, 04/09/18, 04/19/18, 05/04/18	10/20/60 m	(EAS, 2017)
5 Landsat-8	12/05/17, 12/21/17, 02/23/18, 03/27/18, 04/28/18	15/30/60 m	(EAS, 2017)

**Table 3:** Distribution of training and verification pixels for land-cover classes.

Class	Training Pixels	Testing Pixels
Forest	620	729
Maize	568	489
Rape	518	475
Urban	539	510
Water	730	658
Wheat	492	585

**Table 4:** Features extracted from spectral vegetation and texture features.

Feature	Factor	S1	S2	L	Describe
	Mean	/	10	7	Mean of each band (S2: 2–8, 8a, 11–12; L: 1–7)
Spectral	Standard Deviation	/	10	7	Standard deviation of each band (S2: 2–8,8a,11–12; L: 1–7)
	Variance	/	10	7	Variance of each band (S2: 2–8, 8a,11–12; L: 1–7)
	Backscatter coefficient	3	/	/	The S1 Band: VH, VV, CR;
Vegetation Indices	Enhanced Vegetation Index (EVI)	/			2.5*((NIR-R)/(NIR + 6*R − 7.5*B + 1)) [41]
	Normalized Difference Vegetation Index (NDVI)-B8a	/		/	(NIR2-R)/(NIR2 + R) [23]
	(NDVI)-B76	/		/	(RE3-RE2)/(RE3 + RE2) [23]
	(NDVI)-B8a5	/		/	(NIR2-RE1)/(NIR2 + RE1) [23]
	(NDVI)-B65	/		/	(RE2-RE1)/(RE2 + RE1) [23]
	(NDVI)-B75	/		/	(RE3-RE1)/(RE3 + RE1) [23]
	(NDVI)-B8a6	/		/	(NIR2-RE2)/(NIR2 + RE2) [23]
	NDVI	/			(NIR-R)/(NIR + R) [23]
	Triangular Vegetation Index (TVI)	/			0.5(120(NIR-G)-200(R-G)) [42]
	Normalized Difference Water Index (NDWI)	/			(NIR-SWIR1)/(NIR + SWIR1) [43]
	Normalized Difference Tillage Index (NDTI)	/			(SWIR1-SWIR2)/(SWIR1 + SWIR2) [44]
	Mean (ME)	3			Gray-Level Co-occurrence Matrix (GLCM) homogeneity of all directions
	Variance (VA)	3			
	Homogeneity (HO)	3			
Texture	Contrast (CON)	3			
	Dissimilarity (DI)	3			
	Entropy (EN)	3			
	Second moment (SM)	3			
	Correlation (COR)	3			

**Table 5:** Description of the combined vector variables. The S1 represents Sentinel-1, S2 represents Sentinel-2, L represents Landsat-08; T represents Texture feature, S represents Spectral feature, I represents Index feature; TS: Texture + Spectral; TSI: Texture + Spectral + Index.

Sensor	Variables	Description
S1	S1(T)	Textural features of the time series of Sentinel-1 data
	S1(S)	Spectral features of the time series Sentinel-1 data
	S1(TS)	Combined textural and spectral features of the time series of Sentinel-1 data
S2	S2(T)	Textural features of the time series of Sentinel-2 data
	S2(TS)	Combined textural and spectral features of the time series of Sentinel-2 data
	S2(TSI)	Combined textural, spectral, and indices features of the time series of Sentinel-2 data
L	L(T)	Textural features of the time series of Landsat-8 data
	L(TS)	Combined textural and spectral features of the time series of Landsat-8 data
	L(TSI)	Combined textural, spectral and indices features of the time series of Landsat-8 data
S1+L	S1(TS)+L(TSI)	Combine textural and spectral features of Sentinel-1 data and textural, spectral and indices features of Landsat-8 data
S2+L	S2(TSI)+L(TSI)	Combined textural, spectral, and indices features of Sentinel-2 and Landsat-8 data
S1+S2	S1(TS)+S2(TSI)	Combined textural and spectral features of time series of Sentinel-1 data and Sentinel-2 texture, spectral, and indices features
S1+S2+L	S1(TS)+S2(TSI)+L(TSI)	Combination of all three features of each sensor

**Table 6:** The overall accuracy (OA), Kappa coefficient, and *F*1 accuracy of different features with three classifiers from Sentinel-1, Sentinel-2, and Landsat-08 satellite data. RF: Random Forest, SVM: Support Vector Machine, ANN: Artificial Neural Network.

			OA	KP	Forest		Maize		Rape		Urban		Water		Wheat	
					*F*1	*FoM*	*F*1	*FoM*	*F*1	*FoM*	*F*1	*FoM*	*F*1	*FoM*	*F*1	*FoM*
S1	T	RF	0.75	0.70	0.67	0.41	0.66	0.41	0.71	0.53	0.62	0.39	0.87	0.76	0.92	0.85
		SVM	0.70	0.64	0.66	0.43	0.64	0.38	0.66	0.51	0.58	0.39	0.76	0.68	0.90	0.80
		ANN	0.68	0.61	0.50	0.39	0.55	0.42	0.61	0.51	0.53	0.38	0.88	0.78	0.87	0.78
	S	RF	0.71	0.65	0.51	0.35	0.58	0.41	0.70	0.54	0.53	0.37	0.87	0.76	0.88	0.78
		SVM	0.67	0.61	0.51	0.34	0.53	0.36	0.66	0.50	0.45	0.29	0.86	0.76	0.87	0.76
		ANN	0.68	0.62	0.48	0.32	0.57	0.40	0.67	0.51	0.47	0.31	0.87	0.77	0.88	0.79
	TS	RF	0.77	0.72	0.68	0.51	0.68	0.51	0.72	0.57	0.64	0.47	0.87	0.77	0.91	0.84
		SVM	0.73	0.67	0.61	0.44	0.61	0.43	0.68	0.53	0.57	0.40	0.90	0.82	0.90	0.82
		ANN	0.69	0.61	0.54	0.37	0.54	0.37	0.65	0.48	0.51	0.34	0.90	0.82	0.87	0.77
S2		RF	0.86	0.83	0.84	0.73	0.86	0.76	0.84	0.73	0.81	0.67	0.95	0.91	0.88	0.78
	T	SVM	0.85	0.82	0.72	0.57	0.76	0.68	0.85	0.74	0.82	0.70	0.95	0.91	0.93	0.87
		ANN	0.82	0.77	0.61	0.44	0.82	0.70	0.81	0.68	0.83	0.71	0.96	0.93	0.79	0.66
		RF	0.88	0.85	0.84	0.73	0.87	0.77	0.85	0.74	0.85	0.74	0.95	0.91	0.91	0.84
	TS	SVM	0.83	0.80	0.75	0.60	0.80	0.66	0.78	0.67	0.80	0.66	0.90	0.82	0.96	0.93
		ANN	0.80	0.74	0.70	0.54	0.84	0.73	0.76	0.62	0.82	0.70	0.90	0.82	0.89	0.81
		RF	0.91	0.89	0.82	0.69	0.89	0.81	0.87	0.77	0.86	0.76	1.00	0.99	0.96	0.93
	TSI	SVM	0.84	0.80	0.78	0.67	0.85	0.74	0.76	0.62	0.81	0.68	0.96	0.93	0.89	0.81
		ANN	0.85	0.82	0.77	0.63	0.82	0.70	0.80	0.66	0.78	0.67	0.98	0.96	0.92	0.85
L		RF	0.79	0.74	0.75	0.60	0.76	0.68	0.74	0.59	0.77	0.63	0.90	0.82	0.83	0.71
	T	SVM	0.74	0.67	0.64	0.38	0.67	0.51	0.67	0.51	0.72	0.57	0.90	0.82	0.83	0.71
		ANN	0.75	0.69	0.66	0.41	0.67	0.51	0.69	0.53	0.77	0.63	0.88	0.79	0.82	0.70
		RF	0.84	0.81	0.85	0.74	0.82	0.70	0.79	0.66	0.79	0.66	0.95	0.91	0.88	0.79
	TS	SVM	0.80	0.75	0.72	0.57	0.75	0.60	0.73	0.57	0.81	0.68	0.89	0.83	0.89	0.83
		ANN	0.79	0.75	0.72	0.57	0.70	0.54	0.73	0.57	0.79	0.66	0.92	0.85	0.89	0.83
		RF	0.86	0.83	0.80	0.66	0.84	0.73	0.79	0.66	0.87	0.76	0.95	0.91	0.91	0.84
	TSI	SVM	0.81	0.77	0.73	0.59	0.77	0.63	0.75	0.60	0.80	0.66	0.93	0.87	0.89	0.83
		ANN	0.83	0.79	0.79	0.66	0.77	0.63	0.74	0.59	0.85	0.74	0.94	0.89	0.88	0.79

**Table 7:** OA, Kappa, and *F*1 values for the composite S1, S2, and L data with three classifiers. *FoM*: Figure of Merit.

ID		OA	KP	Forest		Maize		Rape		Urban		Water		Wheat	
				*F*1	*FoM*	*F*1	*FoM*	*F*1	*FoM*	*F*1	*FoM*	*F*1	*FoM*	*F*1	*FoM*
S1+L	RF	0.87	0.84	0.79	0.66	0.82	0.70	0.81	0.68	0.83	0.71	0.97	0.94	0.91	0.84
	SVM	0.80	0.78	0.68	0.52	0.75	0.60	0.79	0.66	0.77	0.63	0.92	0.85	0.87	0.76
	ANN	0.81	0.79	0.72	0.57	0.69	0.52	0.80	0.66	0.82	0.69	0.94	0.89	0.92	0.85
S1+S2	RF	0.92	0.90	0.87	0.77	0.91	0.80	0.86	0.76	0.89	0.83	0.99	0.98	0.96	0.93
	SVM	0.84	0.80	0.77	0.63	0.77	0.63	0.78	0.65	0.75	0.61	0.93	0.87	0.95	0.91
	ANN	0.85	0.81	0.79	0.66	0.77	0.63	0.79	0.66	0.80	0.66	0.97	0.94	0.95	0.91
S2+L	RF	0.91	0.88	0.85	0.74	0.93	0.87	0.88	0.79	0.86	0.76	0.98	0.96	0.94	0.89
	SVM	0.85	0.81	0.75	0.60	0.87	0.76	0.83	0.71	0.76	0.62	0.95	0.91	0.92	0.85
	ANN	0.85	0.81	0.74	0.59	0.85	0.74	0.89	0.83	0.71	0.53	0.96	0.93	0.94	0.89
S1+S2+L	RF	0.93	0.91	0.87	0.77	0.94	0.89	0.91	0.80	0.89	0.83	0.99	0.98	0.96	0.93
	SVM	0.85	0.82	0.75	0.60	0.75	0.60	0.84	0.73	0.78	0.64	0.97	0.94	0.96	0.93
	ANN	0.86	0.83	0.84	0.73	0.86	0.76	0.84	0.73	0.81	0.67	0.95	0.91	0.88	0.79

**Table 8:** Confusion Matrix of the best winter crop classifications obtained using a parameter dataset derived from a combination of S1, S2, and L time series. Overall accuracy = 93%, Kappa index = 0.91.

	Forest	Maize	Rape	Urban	Water	Wheat	*U*	*F*1	*FoM*
**Forest**	461	1	31	0	0	2	0.93	0.87	0.77
**Maize**	0	709	11	4	7	0	0.97	0.94	0.89
**Rape**	62	24	1036	62	7	2	0.87	0.89	0.80
**Urban**	0	38	38	690	0	0	0.9	0.91	0.83
**Water**	0	0	4	0	912	0	1.00	0.99	0.98
**Wheat**	42	0	26	0	0	946	0.96	0.98	0.93
***P***	0.82	0.92	0.90	0.91	0.98	1.00			
